# Mitochondrial phylogenomics of Acanthocephala: nucleotide alignments produce long-branch attraction artefacts

**DOI:** 10.1186/s13071-022-05488-0

**Published:** 2022-10-19

**Authors:** Jin-Wei Gao, Xi-Ping Yuan, Hao Wu, Chuan-Yu Xiang, Min Xie, Rui Song, Zhong-Yuan Chen, Yuan-An Wu, Dong-Sheng Ou

**Affiliations:** 1 Hunan Fisheries Science Institute, 728 Shuanghe Rd, Kaifu District, Changsha, 410153 Hunan China; 2 State Key Laboratory of Grassland Agro-Ecosystems and College of Ecology, Lanzhou University, Lanzhou, 730000 China; 3 Hunan Provincial Key Laboratory for Molecular Immunity Technology of Aquatic Animal Diseases, College of Life and Environmental Sciences, Hunan University of Arts and Science, Changde, 415000 Hunan China

**Keywords:** *Micracanthorhynchina dakusuiensis*, Phylogeny, Phylogenetic analysis, Taxonomy, Mitochondrial genome, Rhadinorhynchidae, Echinorhynchida, Compositional heterogeneity

## Abstract

**Background:**

Classification of the Acanthocephala, a clade of obligate endoparasites, remains unresolved because of insufficiently strong resolution of morphological characters and scarcity of molecular data with a sufficient resolution. Mitochondrial genomes may be a suitable candidate, but they are available for a small number of species and their suitability for the task has not been tested thoroughly.

**Methods:**

Herein, we sequenced the first mitogenome for the large family Rhadinorhynchidae: *Micracanthorhynchina dakusuiensis*. These are also the first molecular data generated for this entire genus. We conducted a series of phylogenetic analyses using concatenated nucleotides (NUC) and amino acids (AAs) of all 12 protein-coding genes, three different algorithms, and the entire available acanthocephalan mitogenomic dataset.

**Results:**

We found evidence for strong compositional heterogeneity in the dataset, and *Micracanthorhynchina dakusuiensis* exhibited a disproportionately long branch in all analyses. This caused a long-branch attraction artefact (LBA) of *M. dakusuiensis* resolved at the base of the Echinorhynchida clade when the NUC dataset was used in combination with standard phylogenetic algorithms, maximum likelihood (ML) and Bayesian inference (BI). Both the use of the AA dataset (BI-AAs and ML-AAs) and the CAT-GTR model designed for suppression of LBA (CAT-GTR-AAs and CAT-GTR-NUC) at least partially attenuated this LBA artefact. The results support Illiosentidae as the basal radiation of Echinorhynchida and Rhadinorhynchidae forming a clade with Echinorhynchidae and Pomporhynchidae. The questions of the monophyly of Rhadinorhynchidae and its sister lineage remain unresolved. The order Echinorhynchida was paraphyletic in all of our analyses.

**Conclusions:**

Future studies should take care to attenuate compositional heterogeneity-driven LBA artefacts when applying mitogenomic data to resolve the phylogeny of Acanthocephala.

**Graphical Abstract:**

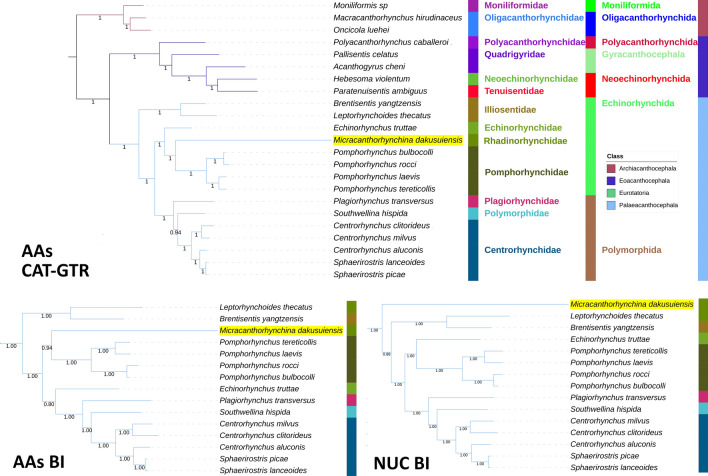

**Supplementary Information:**

The online version contains supplementary material available at 10.1186/s13071-022-05488-0.

## Background

The Acanthocephala or thorny-headed worms is a clade of obligate endoparasites comprising around 1300 species [1] of great veterinary and sometimes even medical importance [2, 3]. Traditionally, the Acanthocephala was a stand-alone phylum, but in more recent classifications it forms a phylum Syndermata together with the Rotifera (Protostomia: Spiralia: Gnathifera: Syndermata) [3, 4]. It is currently divided into three classes: Archiacanthocephala, Palaeacanthocephala, and Eoacanthocephala (recently merged with Polyacanthocephala), with the latter two being sister clades in most molecular analyses [5, 6].

Morphological identification of small parasitic animals is often fraught with difficulties due to the low number of suitable morphologic traits and common host-induced morphological variability [7–9], both of which have been reported in acanthocephalans [10, 11]. Inferring their evolutionary history is further hampered by the fact that fossils are very rare for soft-bodied parasitic animals. It is, therefore, necessary to integrate morphological and molecular data with sufficient resolution for the identification and phylogenetic studies of parasites [12]. Molecular data remain unavailable for many acanthocephalan lineages, and available data are mostly limited to single genes or gene fragments. Such small molecular markers generally have an insufficient resolution for resolving deep evolutionary splits, such as those of syndermatans, whose evolutionary history probably dates back hundreds of millions of years [3]. As a result of this scarcity of molecular data and limited resolution of molecular data, the taxonomy and phylogeny of Acanthocephala remain very poorly resolved despite multiple previous attempts to decipher their evolutionary history [5, 13, 14]. Even the higher level phylogeny remains incompletely resolved. For example, some of the classes appear paraphyletic in some studies based on morphological characters and the *18S* gene [4, 5, 15]. Furthermore, the monophyly of some orders, such as the Echinorhynchida, remains questionable, and the positions and monophyly of many families remain unresolved [1, 3, 5, 16, 17]. Among the multiple unresolved family-level relationships, several studies indicated that the large family Rhadinorhynchidae Lühe, 1912, may be paraphyletic or polyphyletic, so its status remains uncertain [11, 15, 18].

Due to their numerous comparative advantages, most notably unilinear inheritance and absence of recombination, mitochondrial genomes were initially thought to be the ideal molecular marker for studying the evolutionary history of life on earth [19]. Although multiple subsequent studies have shown that issues such as long-branch attraction (LBA) and base composition biases may hamper different types of mitogenomic evolutionary analyses, especially those aimed at deep evolutionary splits [20–23], once these limitations are accounted for, mitogenomes remain an invaluable marker for phylogenetic, taxonomic and evolutionary studies [21, 24]. Previous studies found some limited evidence that different mitochondrial phylogenomics approaches generally produce stable acanthocephalan topologies, but this was largely inferred on the basis of a single dataset, either nucleotide or amino acid sequences of 12 concatenated genes, and two standard phylogenetic inference methods, maximum likelihood and Bayesian inference [5, 16, 25]. As there is evidence that nucleotide and amino acid datasets can produce different topologies [22, 26], both datasets should be tested and topology stability between them assessed. However, none of the previous studies had tested the topological stability thoroughly. Furthermore, mitogenomic sequences of acanthocephalans appear to exhibit high evolutionary rates [13], and compositional heterogeneity was previously identified for the overall syndermatan mitogenomic dataset [3, 13]. As both factors can affect phylogenetic reconstruction [27, 28], it is necessary to thoroughly assess their presence and impacts in this dataset. However, the above studies [3, 13] included a very small number of acanthocephalan mitogenomes, so it remains unknown whether the acanthocephalan dataset also exhibits compositional heterogeneity. Finally, previous studies have not attempted to use phylogenetic inference algorithms designed to attenuate compositional heterogeneity-driven long branch artefacts, such as CAT-GTR [28], which sometimes produces topologies different from the standard phylogenetic inference methods [22, 26]. Therefore, the suitability of mitogenomic data for inferring the evolutionary history of Acanthocephala needs to be tested systematically before they are applied to this task.

The applicability of mitogenomic data is further hampered by the absence or poor representation of many acanthocephalan lineages in terms of publicly available sequenced annotated mitogenomes. There are currently no mitogenomes available for the entire large family, and molecular data remain completely unavailable for the genus *Micracanthorhynchina* (Rhadinorhynchidae). This scarcity of molecular data hampers both evolutionary studies and the identification of acanthocephalan parasites. To address this dearth of data and explore the stability of acanthocephalan topology inferred using mitochondrial phylogenomics, we sequenced the complete mitogenome of *Micracanthorhynchina dakusuiensis* (Harada, 1938) Ward, 1951, and used it to conduct thorough phylogenetic analyses of the entire available acanthocephalan mitogenomic dataset.

## Methods

The parasite was collected on 7 September 2018 from the intestinal tract of the host, yellow catfish *Tachysurus* (syn. *Pelteobagrus*) *fulvidraco* (Richardson, 1846) (Bagridae) sampled from the South Dongting Lake, Yuanjiang county, Hunan province, China (28°50’N, 112°23’E). The mitogenome was sequenced following the methodology described before [26]. Briefly, DNA was isolated from the complete specimen using the AidLab DNA extraction kit (AidLab Biotechnologies, Beijing, China). Primers (Additional file 1: Table S1) used to amplify and sequence the entire mitogenome were designed to match conserved regions of mitochondrial genes of orthologues and to produce overlapping amplicons (≈100 bp). PCR reaction mixture of 50 µl comprised: 5 U µl^−1^ TaKaRa LA Taq polymerase (TaKaRa, Japan), 10 × LATaq Buffer II, 2.5 µM dNTP mixture, 0.2–1.0 µM of each primer, and 60 ng of DNA template. After the initial denaturation at 98 °C for 2 min, 40 PCR cycles comprised 98 °C for 10 s, 50 °C for 15 s, and 68 °C for 1 min kb^−1^. When the product was not specific enough, PCR conditions were optimized by increasing the annealing temperature and decreasing the number of cycles. PCR products were sequenced using the same set of primers and the Sanger method. Electropherograms were visually inspected, and their identity was confirmed using BLAST. The mitogenome was assembled manually using DNASTAR v. 7.1 [29]. By confirming that overlaps were identical, we made sure that the mitogenome is complete, circular, and no *numt*s were incorporated into the sequence. The mitogenome was roughly annotated using MITOS [30], and the annotation was then manually fine tuned. tRNAs were additionally identified using ARWEN [31]. PhyloSuite [32] was used to parse and extract the annotation and generate the file for submission to GenBank.

For the mitogenomic dataset, we downloaded all available Acanthocephala mitogenomes (9 April 2022) and used one per species. Nine mitogenomes were unannotated so they were removed. This left 23 sequences in the dataset. We added the sister clade Rotifera [15] as the outgroup. As many, but not all, Rotifera possess fragmented mitogenomes, we selected species that have complete mitogenomes (i.e. all genes on a single chromosome): *Rotaria rotatoria* (Bdelloidea: Rotifera: Syndermata) [33] and *Philodina citrina* [5]. PhyloSuite was used to retrieve all mitogenomes, standardise annotation, retrieve taxonomic info from the NCBI, extract mitogenomic data, and generate comparative tables. *Leptorhynchoides thecatus* (NC_006892) [34] was attributed to Rhadinorhynchidae in the GenBank, but we manually reassigned it to Illiosentidae following [35], and *Acanthosentis cheni* (KX108947) [36] was renamed to *Acanthogyrus cheni* (*Acanthogyrus (Acanthosentis) cheni* Amin 2005) [1]. For the NCR extraction, the threshold was set to 100 bp. For the phylogenetic analyses, nucleotide sequences of all 12 PCGs were aligned in the codon mode using the accurate G-ins-i strategy in MAFFT [37] and alignments were then refined using MACSE [38]. Aligned genes were concatenated using PhyloSuite, and the optimal evolutionary model and partitioning scheme were inferred using ModelFinder [39] (Additional file 1: Text S1). Phylogenetic analysis was conducted using three different tools: (i) IQ-TREE [40] with 20,000 ultrafast bootstraps [41]; (ii) MrBayes (RRID: SCR_012067) [42] with default parameters: number of runs = 2; number of chains = 4; burnin = 25%; it was allowed to run until the standard deviation of split frequencies plateaued at values < 0.01 (a very good indication of convergence according to MrBayes manual) (Additional file 1: Text S2); ModelFinder output was used to set the data partitioning and select evolutionary models for both of the above analyses; (iii) PhyloBayes-MPI 1.7a [28], with the CAT-GTR model and default parameters (burnin = 500, invariable sites automatically removed from the alignment, two MCMC chains). The conditions considered to indicate a good run were: maxdiff < 0.1 and minimum effective size > 300.

For the three single-gene datasets (*cox1*, *18S*, *28S*) we downloaded all available Acanthocephala homologues, pruned all sequences that were too short or misaligned, and left only one or two sequences per species, apart from the Rhadinorhynchidae, for which we left all sequences that could be aligned. The final datasets comprised 96 *18S* sequences (11 Rhadinorhynchidae), 174 *28S* sequences (12 Rhadinorhynchidae), and 157 *cox1* sequences (12 Rhadinorhynchidae). Phylogenetic analyses were conducted using IQ-TREE as described above, except for the fact that sequences were aligned only using MAFFT and not in the codon mode. iTOL [40] was used to visualise the phylogeny and architecture using files generated by PhyloSuite.

## Results

### Identity and morphology

**Fig. 1:**
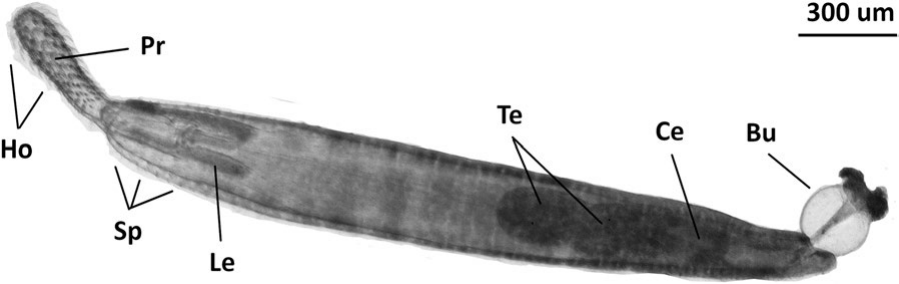
A microscopic image of a *M. dakusuiensis* specimen (male). Visible are proboscis (Pr), proboscis hooks (Ho), spines (Sp), lemniscus (Le), testes (Te), cement gland (Ce), and copulatory bursa (Bu)

Acanthocephalan parasites were collected from the intestinal tract of the host yellow catfish (*Pelteobagrus fulvidraco*) and identified as *Micracanthorhynchina dakusuiensis* on the basis of their morphology [43]. The full taxonomic identity is therefore Palaeacanthocephala (Class): Echinorhynchida (Order): Rhadinorhynchidae (Family): Gorgorhynchinae (Subfamily). The sampled parasites were small (2.6–6.0 mm), with a fusiform trunk (Fig. 1). Anteriorly, the trunk ends with multiple small spines arranged in 8–9 rows all around the trunk, with the last 6–7 rows present only ventrally. The proboscis is large and cylindrical, with 12 circular rows of 9–10 hooks each. The lemniscus is a bit longer than the receptacle of the proboscis. The specimen pictured herein was male, with two oval tandemly arranged testes visible in the middle of the body. There are six clavate cement glands, and the copulatory bursa is reversible.

**Fig. 2:**
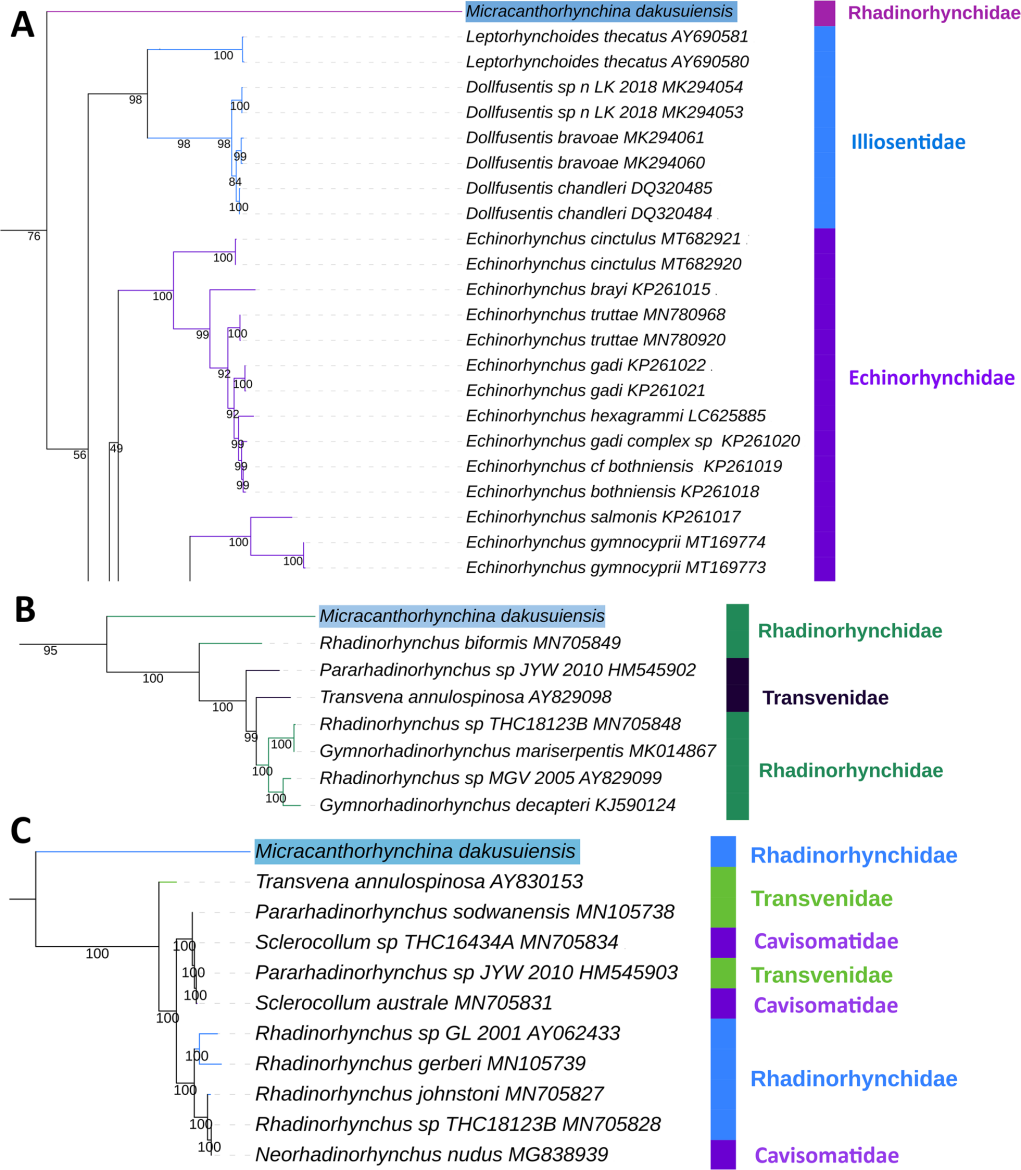
Single gene-based identification of *M. dakusuiensis*: **A** *cox1*, **B** *28S*, **C** *18S*. Only the relevant fragments of phylograms (containing the query sequence) are shown

Due to the absence of molecular data for the entire genus, it was impossible to precisely identify the species molecularly. The barcode (*cox1*) analysis produced a disproportionately long branch of *M. dakusuiensis* and resolved it at the base of the order Echinorhynchida (Fig. 2A). The family Rhadinorhynchidae was deeply paraphyletic. The two nuclear single-gene markers (*18S* and *28S*) identified it with some confidence as a Rhadinorhynchidae species; i.e. it clustered with some of the included Rhadinorhynchidae species (Fig. 2B, C). In both analyses, a fraction of Rhadinorhynchidae comprising *M. dakusuiensis* formed a clade with a fraction of Transvenidae and Cavisomatidae species. However, these and many other families were paraphyletic in both analyses*.*

### Comparative mitogenomic architecture

**Fig. 3:**
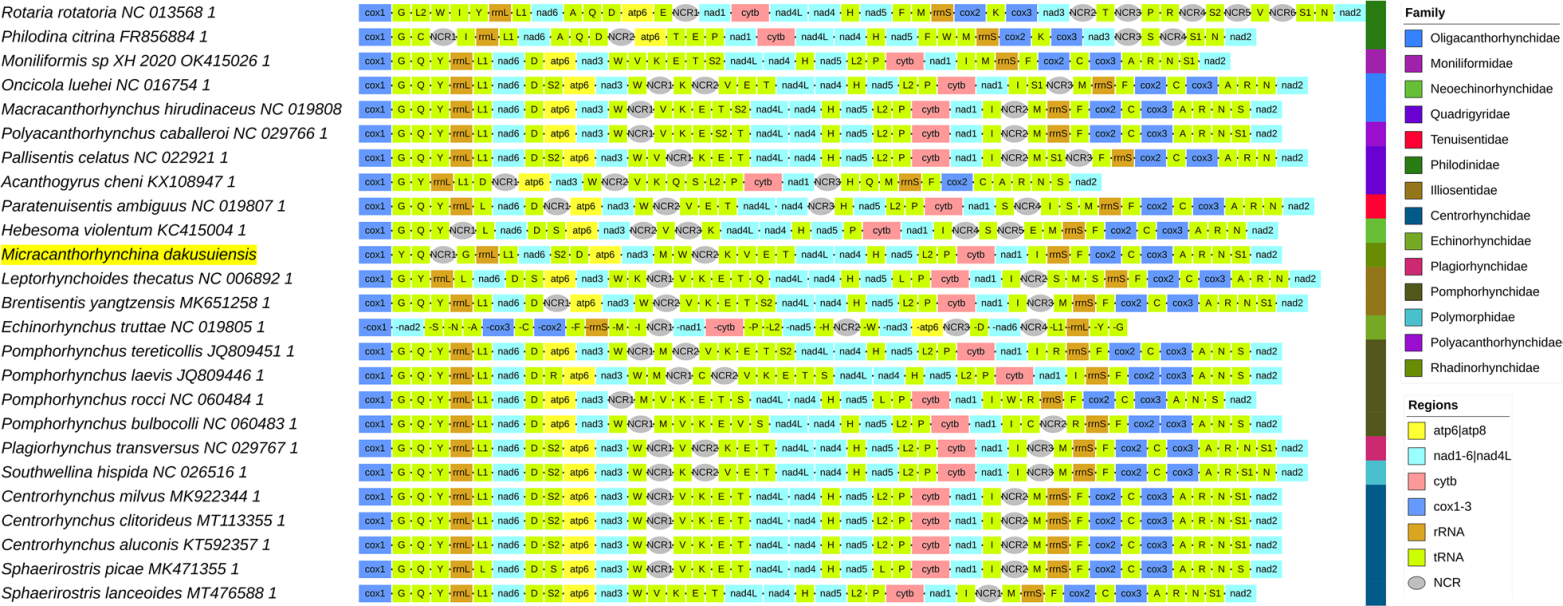
Gene orders of *M. dakusuiensis* and the remaining available Acanthocephala mitogenomes as well as two Rotifera outgroups. The legend is shown in the figure, species names are given with corresponding GenBank accession numbers, and the family-level taxonomic identity is shown to the right. NCR indicates an intergenic region > 100 bp

The mitogenome of *M. dakusuiensis* had a circular structure with all 36 genes encoded on the same strand. *Atp8* was not identified. At 16,309 bp, the size of the mitogenome was relatively large within the acanthocephalan dataset, which ranged in size from 13,393 to 15,884 bp (Additional file 2: Dataset S1). There were two large non-coding regions (NCR), 1470 bp and 2123 bp, while other NCRs were all < 40 bp in size (Additional file 1: Table S2). Start codons of protein-coding genes (PCGs) were standard: the most common was GTG (8 genes), followed by TTG (2 genes) and ATG (2). Stop codons were also standard: TAG (6 genes), TAA (3 genes), and the abbreviated T– (3 genes). Many genes were remarkably divergent within the dataset, with very few conserved segments, multiple non-alignable segments, and a wide range of gene sizes. *nad4L* was so highly divergent within the dataset that there were only eight sites conserved among all species included. The genes of *M. dakusuiensis* did not exhibit extreme size values, apart from *nad4*: due to several deletions, it spanned only 1197 bases, which made it the smallest among the orthologues included in the dataset. This is not sufficient to suspect an annotation artefact, as the second smallest orthologue was only 10 bp larger, and the size range among the remaining orthologues was very wide (1207 to 1308 bases) (Additional file 2: Dataset S2). In terms of gene order, the newly sequenced *M. dakusuiensis* exhibited a large number of tRNA rearrangements in comparison to all available acanthocephalan mitogenomes, but the variability was limited to tRNA genes and large NCRs (Fig. 3).

Regarding the base composition, the A + T content range was wide in the dataset (54.5–71.4%), with *M. dakusuiensis* in the lower end of the range (56.8%). The GC skew (on the entire coding strand) was 0.43, which is slightly below the average for the Acanthocephala (0.39–0.68 in the currently available dataset), whereas the AT skew was − 0.25, which was an average value in the dataset (− 0.17 to − 0.35) (Additional file 2: Dataset S1).

### Phylogeny

**Fig. 4:**
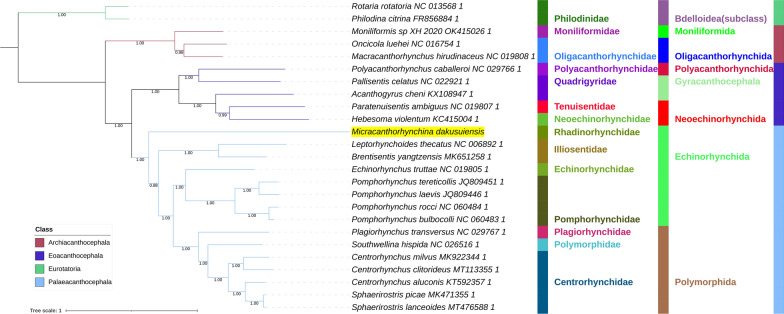
The NUC—BI phylogeny of Acanthocephala. The analysis was conducted using concatenated and partitioned nucleotide sequences of all 12 mitogenomic PCGs. BI is Bayesian inference as implemented in MrBayes. Support values are shown at corresponding nodes. All mitogenomes are shown with GenBank accession numbers, and the newly sequenced *M. dakusuiensis* is highlighted in yellow. The family, order and class-level taxonomic identities are shown to the right

**Fig. 5:**
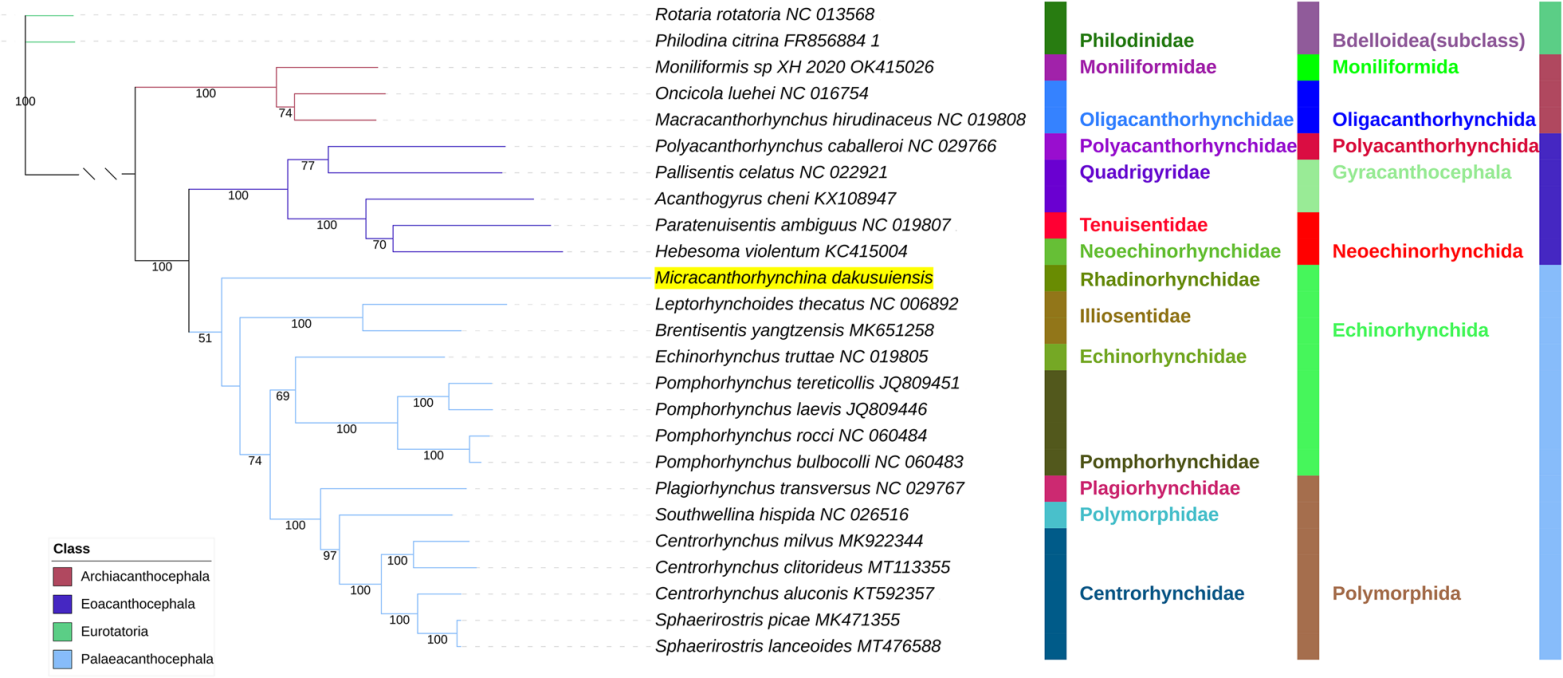
The NUC—ML phylogeny of Acanthocephala. The analysis was conducted using concatenated and partitioned nucleotide sequences of all 12 mitogenomic PCGs. ML is maximum likelihood as implemented in IQ-TREE. See Fig. 4 for other details

**Fig. 6:**
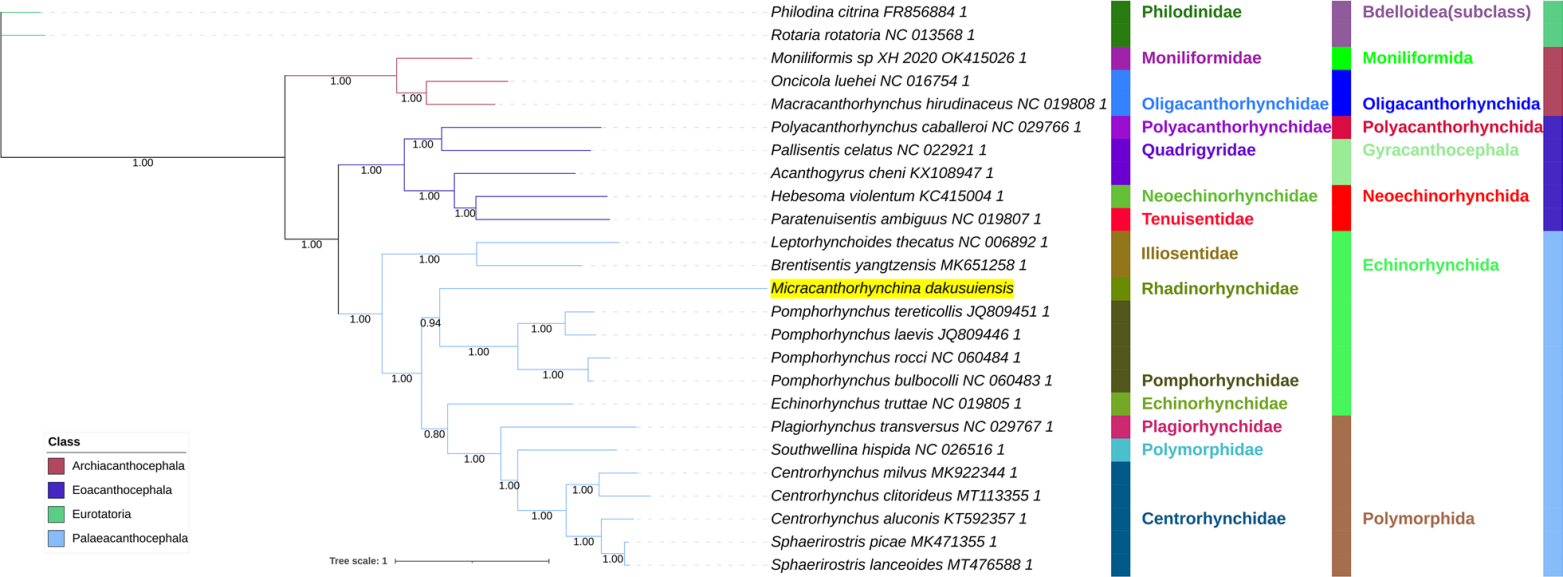
The AAs—BI phylogeny of Acanthocephala. The analysis was conducted using concatenated and partitioned amino acid sequences of all 12 mitogenomic PCGs. BI is Bayesian inference as implemented in MrBayes. See Fig. 4 for other details

**Fig. 7:**
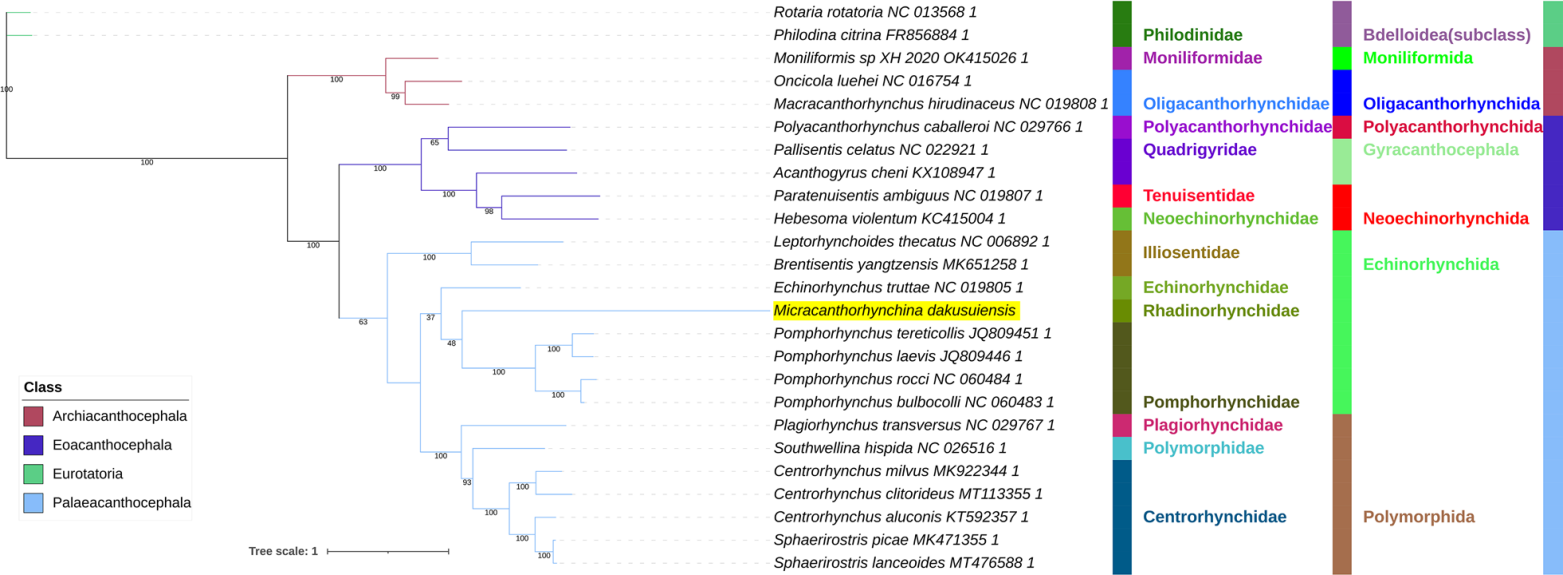
The AAs—ML phylogeny of Acanthocephala. The analysis was conducted using concatenated and partitioned amino acid sequences of all 12 mitogenomic PCGs. ML is maximum likelihood as implemented in IQ-TREE. See Fig. 4 for other details

**Fig. 8:**
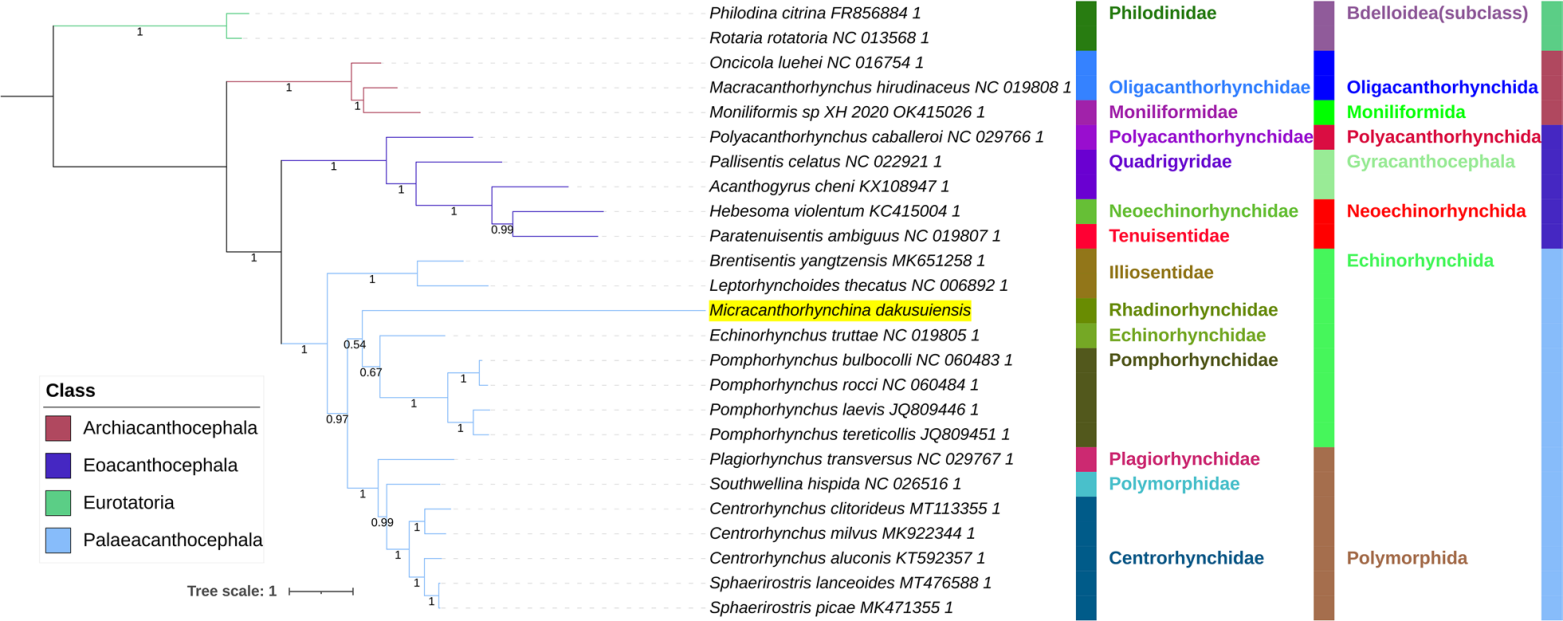
The NUC—CAT-GTR phylogeny of Acanthocephala. The analysis was conducted using concatenated and partitioned nucleotide sequences of all 12 mitogenomic PCGs. See Fig. 4 for other details

**Fig. 9:**
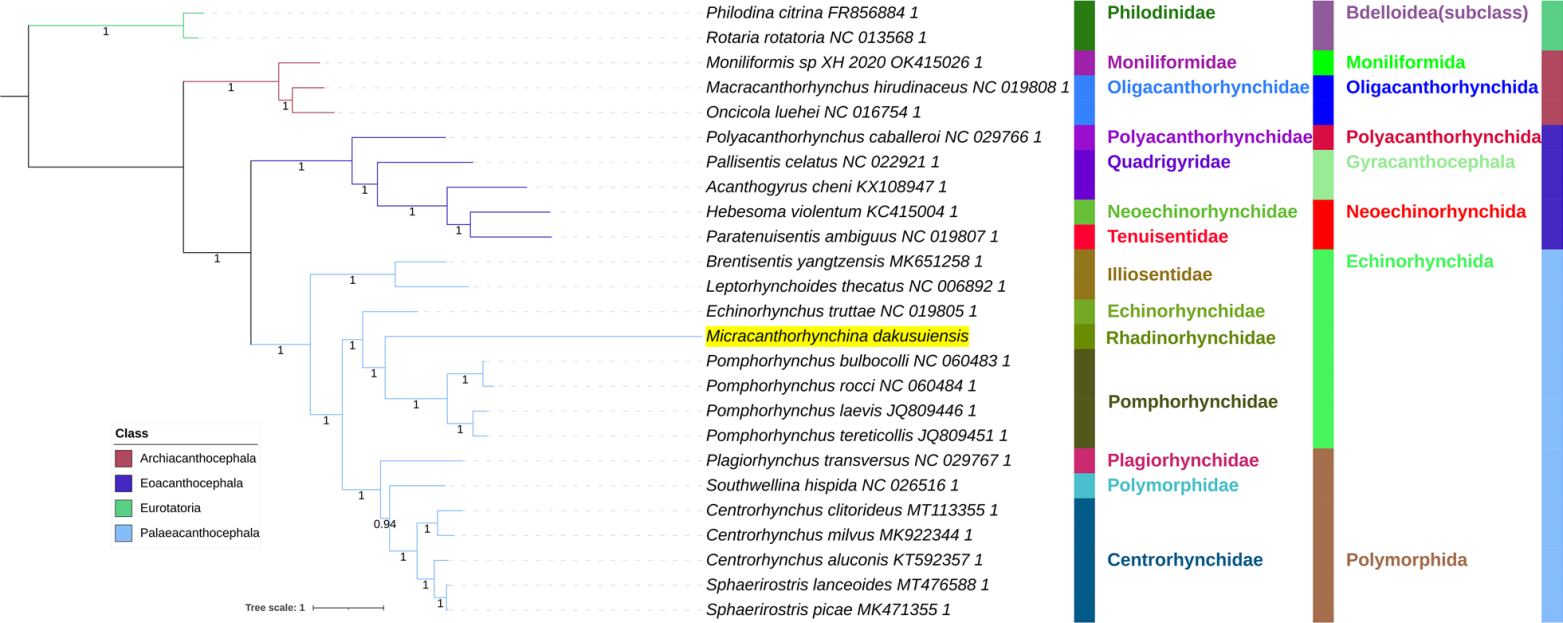
The AAs—CAT-GTR phylogeny of Acanthocephala. The analysis was conducted using concatenated and partitioned amino acid sequences of all 12 mitogenomic PCGs. See Fig. 4 for other details

All included NUC sequences failed the compositional homogeneity Chi-square test implemented in IQ-TREE. In the AA dataset, only *Plagiorhynchus transversus* passed the test (*P* = 7.32%). These results indicate the existence of a very strong compositional heterogeneity in both datasets. BI and ML (IQ-TREE) analyses produced identical NUC topologies and very similar AAs topologies, but there were major differences between the NUC and AAs datasets (Figs. 4, 5, 6, 7). Differences in topologies were almost exclusively caused by the varying position of the newly sequenced *M. dakusuiensis*. Both NUC dataset analyses (BI and ML) produced a topology where *M. dakusuiensis* was at the base (used in the sense: sister group to all other lineages) of Palaeacanthocephala (Figs. 4, 5). In the BI topology, most support values, including the support for this node, were 1.0. In the ML topology, several node support values were low, which included only 51% for the *M. dakusuiensis* node. This topology rendered Echinorhynchida and Rhadinorhynchidae paraphyletic (*Centrorhynchus* aside, all other taxa were monophyletic). The AAs dataset (both BI and ML analyses) produced topologies where Illiosentidae were at the base of Palaeacanthocephala (Figs. 6, 7). In the ML analysis, *M. dakusuiensis* formed a clade with Echinorhynchidae and Pomporhynchidae: [Echinorhynchidae + (*M. dakusuiensis* + Pomporhynchidae)]. In the BI topology, *M. dakusuiensis* formed a clade with Pomporhynchidae, whereas Echinorhynchidae (*Echinorhynchus truttae*) was a sister lineage to the Polymorphida clade. In both analyses, Echinorhynchida and Rhadinorhynchidae were again paraphyletic taxa. In the BI analysis, all support values were 1.0. In the ML topology, several node support values were again low, including only 48% for the *M. dakusuiensis* branch. Both CAT-GTR analyses (NUC and AAs) produced topologies more closely resembling the AAs than the NUC topologies of the other two algorithms (Figs. 8, 9). Both analyses produced maxdiff values that indicate a good run (AAs = 0.065; NUC = 0.061), and the minimum effective size values remained between 50 and 300, which indicated an acceptable run. The CAT-GTR-AAs topology was identical to the ML-AAs topology. The CAT-GTR-NUC topology was also very similar to the ML-AAs topology, but the topology of the variable clade was changed: (*M. dakusuiensis* + (Echinorhynchidae + Pomporhynchidae)). Both topologies exhibited 1.0 support values for almost all nodes. The topology of the remaining two classes was stable, with the Archiacanthocephala as the sister group to the remaining acanthocephalans in all topologies. To assess whether the inclusion of distantly-related taxa affected the topology, we conducted ML analyses of only the Palaeacanthocephala dataset, with one representative of Eoacanthocephala and Archiacanthocephala each as outgroups. The topologies were identical to the corresponding complete acanthocephalan NUC-ML AAs-ML topologies (Additional file 1: Figs S1 and S2).

## Discussion

The mitogenome of *M. dakusuiensis* exhibited the standard acanthocephalan architecture, including the features of missing *atp8* and all 36 genes encoded on the same strand [2, 44, 45]. The average mitogenome size is relatively small in the Syndermata [44], so at 16,309 bp, *M. dakusuiensis* possessed the largest mitogenome in the dataset. Somewhat surprisingly, it exhibited only two NCRs larger than 100 bp, whereas several species with smaller mitogenomes exhibited three to five such NCRs (Fig. 3). However, both NCRs of *M. dakusuiensis* were > 1 Kbp, which is exceptional within the dataset. We inspected the base composition in the dataset, as there is strong evidence that base composition biases can affect phylogenetic and other evolutionary analyses [22, 23, 27]. The newly sequenced *M. dakusuiensis* exhibited a relatively low A + T content within the dataset, but none of the base composition parameters was an outlier within the dataset. Although skews were apparently inverted in *E. truttae* (NC_019805) (AT = 0.29, GC = − 0.44), this was an artefact caused by the authors submitting the minus strand to the GenBank, as reflected in all genes being apparently encoded on the minus strand (Fig. 3). While *M. dakusuiensis* was not exceptional in this aspect, the entire dataset exhibited a significant compositional heterogeneity, which is a recognised problem for phylogenetic reconstruction [27].

We conducted multiple phylogenetic analyses using several different datasets and algorithms. Rhadinorhynchidae was paraphyletic in all three single-gene marker analyses, which is in agreement with previous reports that Rhadinorhynchidae may be paraphyletic or polyphyletic [15, 18]. However, we cannot treat this result as reliable because all three markers produced deeply unorthodox phylogenies with numerous paraphyletic taxa, which is indicative of an insufficient resolution of small molecular markers for inferring the evolutionary history of such an old clade. This is indirectly supported by the fact that mitogenomes (12 PCGs) produced much more stable and orthodox topologies. As this is the first sequenced mitogenome for Rhadinorhynchidae, mitogenomic data cannot provide any evidence concerning the monophyly of Rhadinorhynchidae. However, all analyses based on mitogenomic data, including the barcode (*cox1*) analysis, indicated that *M. dakusuiensis* possesses an exceptionally long branch. Disproportionately fast mitogenomic evolution has been associated with parasitism [46, 47] and low locomotory capacity [48] previously, but the entire clade Acanthocephala is parasitic and exhibits low locomotory capacity, so this does not explain the disproportionately long branch of *M. dakusuiensis*. We can only speculate that this species, or possibly a wider lineage, may have gone through a severe population bottleneck in its evolutionary history, causing elevated evolutionary rates [49]. Regardless, we can infer with relative confidence [28] that this caused LBA artefacts in all NUC-based analyses. As a result, in the *cox1* dataset, it did not cluster with other sequences from the same family. Aside from the exceptionally fast mitogenomic evolution in this lineage, we should also not discount the possibility of a mitochondrial introgression event in its evolutionary history, nor the possibility that Rhadinorhynchidae is paraphyletic. Future mitogenomic studies should therefore seek agreement from nuclear genome data to precisely narrow down the underlying cause for this phenomenon.

Mitochondrial phylogenomic analyses revealed that there was notable variability between the NUC and AAs datasets and congruence between the two standard phylogenetic algorithms (ML and BI). Remarkably, both datasets (AAs and NUC) produced topologies closely resembling the AA dataset (ML and BI) when we applied the algorithm designed to account for compositional heterogeneity, CAT-GTR. As *M. dakusuiensis* had an exceptionally long branch in all analyses, this is strongly indicative of the NUC-BI and NUC-ML analyses producing LBA artefacts. The CAT-GTR algorithm can to an extent suppress LBA artefacts, especially in combination with slower-evolving amino acid sequences [22, 26, 28, 50]. On this basis, and the fact that ML-AAs and CAT-GTR-AAs analyses produced congruent topologies, we propose that CAT-GTR topology is the most reliable. A previous *18S*-based analysis found that the earliest diverging clades of the Echinorhynchida are Illiosentidae and Rhadinorhynchidae [15]. Our analyses indicate that this was probably an LBA caused by the long branch of Rhadinorhynchidae and support only Illiosentidae as the basal radiation of Echinorhynchida. This agrees with a previous result produced by the *18S* + *28S* dataset [51]. Due to the poor representation of acanthocephalan lineages, our analyses also cannot resolve the question of the sister lineage to Rhadinorhynchidae. Some previous studies (*18S* and *28S*-based) suggested that Transvenidae is the sister family [52], but another *18S-*based study indicated that Rhadinorhynchidae are deeply paraphyletic and that the sister group relationship with Transvenidae is limited to one of the three Rhadinorhynchidae lineages [18]. As mitogenomes remain unavailable for Transvenidae, and this is the first mitogenome for Rhadinorhynchidae, we cannot assess this hypothesis. On the basis of lineages available in our analyses, Rhadinorhynchidae, or the segment of Rhadinorhynchidae comprising the genus *Micracanthorhynchina* if the family is paraphyletic, most likely forms a clade with Echinorhynchidae and Pomporhynchidae. A similar clade (comprising *Rhadinorhynchus lintoni* and *Rhadinorhynchus pristis*) was observed in the above-mentioned *18S* analysis [18]. As the topology is supported by both nuclear and mitogenomic data, this presents solid evidence in support of this scenario.

Classes were all monophyletic, and their relationships were Archiacanthocephala + (Eoacanthocephala + Palaeacanthocephala), which corresponds to multiple previous studies [5, 17, 25, 51]. All topologies support the synonymization of Eoacanthocephala and Polyacanthocephala [6], but it should be noted that this synonymization should be further confirmed using suitable nuclear data because mitonuclear discordance has been observed in numerous lineages [22, 53]. The clustering of *Centrorhynchus aluconis* with *Sphaerirostris* species has been observed before [17]. These authors argued that this challenges the validity of the genus *Sphaerirostris*. Indeed, the paraphyly can be resolved by merging *Sphaerirostris* and *Centrorhynchus*, but it should be noted that it can also be resolved by renaming *C. aluconis* to *Sphaerirostris aluconis,* thus not challenging the status of any of these genera*.* Before any taxonomic revision can be made, this needs to be confirmed by nuclear data as well. The order Echinorhynchida was paraphyletic in all of our analyses. The paraphyly of Echinorhynchida was previously observed by several other mitogenome-based studies [6, 16, 17, 54] and a nuclear data-based study (*18S* and *28S*) [52]. In this light, our results suggest that these were probably not LBA artefacts. Based on the topology accepted as the most likely in our study, to resolve the paraphyly, it would be necessary to either merge Echinorhynchida and Polymorphida into a single order or place Illiosentidae into a separate order. Before any changes can be proposed, this needs to be supported by studies with all relevant lineages represented as well as by independent nuclear data-based analyses. Regarding other orders, Gyracanthocephala was also paraphyletic due to the Neoechinorhynchida resolved as a derived clade within the Gyracanthocephala. The paraphyly of this order was also observed before [16, 17, 54], but the specific topology was affected by the algorithm and dataset in our study, so future studies should take this instability into consideration.

## Conclusions

In this study, we sequenced the first mitogenome for the large family Rhadinorhynchidae, *M. dakusuiensis*, thereby also generating the first molecular data for this genus. Mitochondrial phylogenomic analyses revealed that there was notable variability between the NUC and AAs datasets and notable congruence between the two standard phylogenetic algorithms (ML and BI). The newly sequenced species exhibited a disproportionately long branch in all analyses, which caused an LBA (*M. dakusuiensis* at the base of the Echinorhynchida clade) when the NUC dataset was used in combination with standard phylogenetic algorithms. The use of the AA dataset and CAT-GTR model designed for suppression of LBA successfully attenuated the major LBA artefact, but some minor topological instability remained. Our analyses thus support Illiosentidae as the basal radiation of Echinorhynchida, whereas Rhadinorhynchidae, or the segment of Rhadinorhynchidae comprising the genus *Micracanthorhynchina* if the family is paraphyletic, most likely forms a clade with Echinorhynchidae and Pomporhynchidae. Due to the scarcity of data for Rhadinorhynchidae and other closely related lineages, such as the Transvenidae, it is impossible to resolve the question of monophyly of Rhadinorhynchidae and identify the sister lineage to this family. There is strong evidence for paraphyly of the order Echinorhynchida, but this needs to be supported by high-resolution nuclear data as well. Mitochondrial genomes are a promising marker for studying the phylogeny of Acanthocephala, but only in combination with methodological approaches that attenuate the compositional heterogeneity-driven LBA artefacts and putative removal of rogue lineages. Future studies should not rely solely on nucleotide sequences and standard phylogenetic methods when applying mitogenomic data to resolve the phylogeny of Acanthocephala.

## Supplementary Information

**Additional file 1**: **Table S1**. Primers used for the amplification and sequencing of the mitogenome of *M. dakusuiensis*. **Table S2**. The architecture of the mitogenome of *M. dakusuiensis*. **Text S1**. Evolutionary models and partitions.** Figure S1**. The NUC—ML phylogeny of Palaeacanthocephala. **Figure S2**. The AAs—ML phylogeny of Palaeacanthocephala. **Text S2**. BI and CAT-GTR analyses run parameters.

**Additional file 2**: **Dataset S1**. Comparative overall mitogenomic architecture features of the Acanthocephala. **Dataset S2**. Comparative features of individual genes in mitogenomes of the Acanthocephala.

## Data Availability

GenBank accession numbers of newly generated sequences are: OP131911 (mitogenome), OP133175 (*18S*), and OP133174 (*28S*).

## Abbreviations

AAs: Amino acid sequences of 12 concatenated PCGs
BI: Bayesian inference
Kbp: Kilo base pair
LBA: Long-branch attraction
ML: Maximum-likelihood
NCR: Non-coding region
NUC: Nucleotide sequences of 12 concatenated PCGs
PCG: Protein-coding gene
