# Supplementary material for: Mitochondrial phylogenomics of Acanthocephala: nucleotide alignments produce long-branch attraction artefacts
Source: Parasit Vectors. 2022 Oct 19;15:376. doi: 10.1186/s13071-022-05488-0 (PMC9583589; doi:10.1186/s13071-022-05488-0)
Supplement: Supplementary file 1 — Additional file 1: Table S1. Primers used for the amplification and sequencing of the mitogenome of M. dakusuiensis. Table S2. The architecture of the mitogenome of M. dakusuiensis. Text S1. Evolutionary models and partitions. Figure S1. The NUC—ML phylogeny of Palaeacanthocephala. Figure S2. The AAs—ML phylogeny of Palaeacanthocephala. Text S2. BI and CAT-GTR analyses run parameters. [file 13071_2022_5488_MOESM1_ESM.pdf]

Additional file 1

for

Mitochondrial phylogenomics of Acanthocephala: nucleotide alignments produce long-branch attraction artefacts

Jin-Wei Gao, Xi-Ping Yuan, Hao Wu, Chuan-Yu Xiang, Min Xie, Rui Song, Zhong-Yuan Chen, Yuan-An Wu, Dong-Sheng Ou

Correspondence: ryain1983@163.com

Table S1. Primers used for the amplification and sequencing of the mitogenome of *M. dakusuiensis*.

Fragment No.	Gene or region	Primer name	Sequence (5'-3')	Length (bp)
F1	*16S*	XJWF1	GTGTAGCGTGATAAGTAGGC	315
		XJWR1	GGTCTAAACTCAGATCACGT	
F2	*16S-CYTB*	XJWF2	GGTAATTACCTTGTAGGGTA	7869
		XJWR2	CTTAGGGAGCTGACCCTAGC	
F3	*CYTB*	XJWF3	TGGTGGTATTACTGATGGTG	471
		XJWR3	GGTACCACTCAGGTTTGATG	
F4	*CYTB-ND1*	XJWF4	GTGTTGTGGGCCCCATACGC	552
		XJWR4	ACTCCATCTAATAGGGGTTG	
F5	*ND1*	XJWF5	GGAGCGTAAAGTGTTAGGTT	554
		XJWR5	CCCTTACTAACTCTGACTCC	
F6	*ND1-12S*	XJWF6	GGGAAGGTGTGGCGTGCG	753

		XJWR6	GCTGTATTACGCAGGTATC	
F7	*12S*	XJWF7	GTTAAGTTCGTGTAGTTTAC	397
		XJWR7	TTGACGGGCGATATGTACTC	
F8	*12S-COX2*	XJWF8	CTGAGTCATAGGTGTAATAC	696
		XJWR8	CAATCCCAGGGATGCAGTC	
F9	*COX2*	XJWF9	GTTTTGCTTGGAGGACTCTT	245
		XJWR9	CACACAGCTCATAACACTGC	
F10	*COX2-16S*	XJWF10	AGGTTGACTGCATCCCTGG	5704
		XJWR1	GTCTTATAACACCCTCAAGC	

Table S2. The architecture of the mitogenome of *M. dakusuiensis*. IGN is intergenic region.

**Gene**	**Position**		**Size**	**IGN**	**Codon**		**Anti-codon**
	**From**	**To**			**Start**	**Stop**	
cox1	1	1533	1533		GTG	TAA	
trnY	1534	1590	57				GTA
trnQ	1591	1641	51				TTG
trnG	3112	3162	51	1470			TCC
rrnL	3163	4075	913				
trnL1	4076	4126	51				TAG
nad6	4127	4553	427		GTG	T	
trnS2	4567	4617	51	13			TGA
trnD	4618	4687	70				GTC
atp6	4705	5223	519	17	TTG	TAG	
nad3	5211	5556	346	-13	GTG	T	

trnM	5589	5643	55	32			CAT
trnW	5616	5669	54	-28			TCA
trnK	7793	7852	60	2123			TTT
trnV	7862	7920	59	9			TAC
trnE	7911	7961	51	-10			TTC
trnT	7960	8018	59	-2			TGT
nad4L	8030	8305	276	11	GTG	TAG	
nad4	8307	9503	1197	1	TTG	TAG	
trnH	9496	9545	50	-8			GTG
nad5	9546	11186	1641		ATG	TAA	
trnL2	11161	11212	52	-26			TAA
trnP	11197	11266	70	-16			TGG
cytb	11269	12378	1110	2	GTG	TAG	
nad1	12368	13259	892	-11	ATG	T	
trnI	13263	13331	69	3			GAT
rrnS	13332	13889	558				
trnF	13890	13950	61				GAA
cox2	13922	14548	627	-29	GTG	TAG	
trnC	14535	14590	56	-14			GCA
cox3	14569	15273	705	-22	GTG	TAA	
trnA	15272	15324	53	-2			TGC
trnR	15304	15371	68	-21			TCG
trnN	15358	15402	45	-14			GTT
trnS1	15399	15444	46	-4			GCT
nad2	15445	16308	864		GTG	TAG	

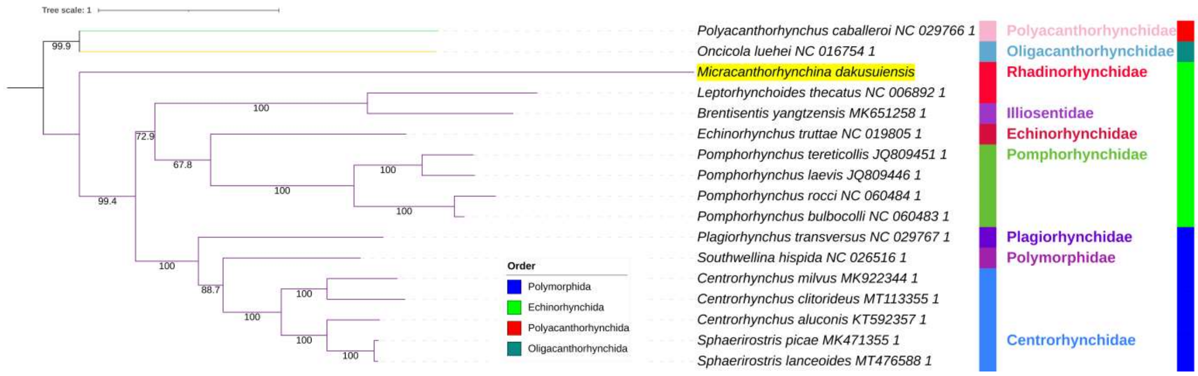

Figure S1. The NUC - ML phylogeny of Palaeacanthocephala. The analysis was conducted using concatenated and partitioned nucleotide sequences of all 12 mitogenomic PCGs. ML is Maximum Likelihood as implemented in IQ-TREE. Family and order-level taxonomic identity is shown in the figure.

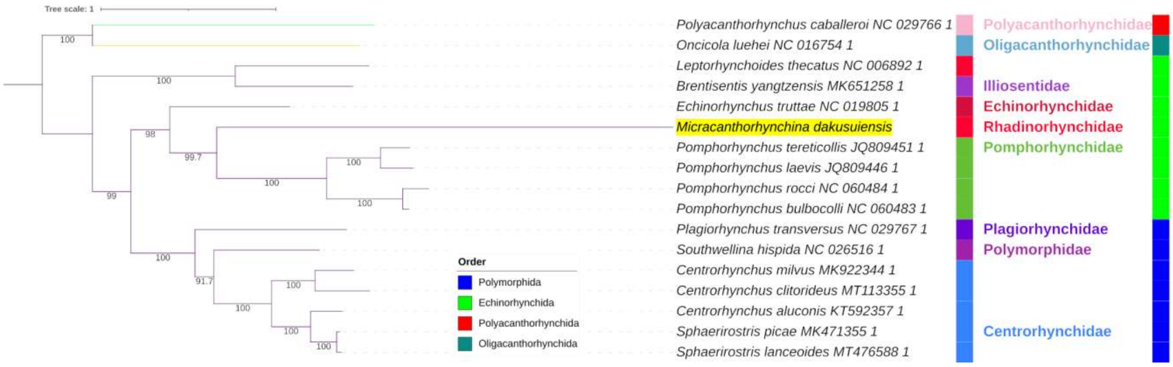

Figure S2. The AAs - ML phylogeny of Palaeacanthocephala. The analysis was conducted using concatenated and partitioned amino acid sequences of all 12 mitogenomic PCGs. ML is Maximum Likelihood as implemented in IQ-TREE. Family and order-level taxonomic identity is shown in the figure.

Text S1. Evolutionary models and partitions.

NUC dataset: partition 1 - *atp6, cox3, nad2, nad3* (model = TVM+F+R4); partition 2 - *cox1* (TVM+F+I+G4); partition 3 - *cox2, cytb, nad1* (GTR+F+I+G4); partition 4 - *nad4L, nad4, nad5, nad6* (GTR+F+R4).

AAs dataset: partition 1 - *cox1, cox2, cytb, nad4L*; partition 2 - all other genes. The optimal model for both partitions was VT+F+I+G4.

Text S2. BI and CAT-GTR analyses run parameters.

NUC-BI: The average standard deviation of split frequencies plateaued after about 150,000 MCMC generations at ≈0.001. As values below 0.01 are a very good indication of convergence, the analysis was allowed to continue till about 750,000 generations before the final tree was inferred.

AAs-BI: When the analysis was interrupted, the value of the average standard deviation of split frequencies had been approximately close to 0.003 (below 0.01), and PSRF (potential scale reduction factor) approached 1, suggesting that convergence had been reached.

NUC-CAT-GTR: The analysis was stopped when the value of maxdiff was 0.061 (below 0.1), which is considered to indicate a good run. The effective size values were in the range between 67 and 1092. As all values were greater than 50, this indicates an acceptable run (according to the PhyloBayes manual).

AAs-CAT-GTR: The analysis was stopped when the value of maxdiff was 0.065 (good run), and effective size values were all in the range 67 to 1510 (acceptable run).
